# Symmetry-breaking antibody binding reveals extensive capsid malleability in pandemic human norovirus

**DOI:** 10.21203/rs.3.rs-9488000/v1

**Published:** 2026-09-21

**Authors:** Carmen V. Apostol, Ramakrishnan Anish, Son Pham, Khalil Ettayebi, Janam Dave, Frederick H. Neill, Marina Bok, Banumathi Sankaran, Sue Crawford, Robert L. Atmar, James E. Crowe, Viviana Parreño, Mary K. Estes, B. V. Venkataram Prasad

**Affiliations:** 1 The Verna and Marrs McLean Department of Biochemistry and Molecular Pharmacology, Baylor College of Medicine, Houston, TX, USA.; 2 Department of Molecular Virology and Microbiology, Baylor College of Medicine, Houston, TX, USA.; 3 Department of Medicine-Gastroenterology and Hepatology, Baylor College of Medicine, Houston, TX, USA.; 4 Incuinta, Instituto Nacional de Tecnología Agropecuaria (INTA), Buenos Aires, ZC, Argentina.; 5 Instituto de Virología e Innovaciones Tecnológicas, Consejo Nacional de Investigaciones Científicas y Técnicas (IVIT-CONICET), Buenos Aires, ZC, Argentina.; 6 Berkeley Center for Structural Biology, Molecular Biophysics and Integrated Bioimaging, Lawrence Berkeley Laboratory, Berkeley, CA, USA.; 7 Department of Pathology, Microbiology and Immunology, Vanderbilt University Medical Center, Nashville, TN, USA.; 8 The Vanderbilt Center for Antibody Therapeutics, Vanderbilt University Medical Center, Nashville, TN, USA.; 9 Department of Pediatrics, Vanderbilt University Medical Center, Nashville, TN, USA.

## Abstract

Virus capsids are metastable assemblies with an exquisite balance between stability and flexibility, obligatory for entry, infectivity, and immune escape. Yet, the extent to which human viruses exploit capsid plasticity and how that consequently shapes antigenic landscapes remains poorly understood. Human noroviruses (HuNoVs) are the leading cause of acute viral gastroenteritis worldwide^1^. GII.4 HuNoVs have been evolving epochally, driving most global pandemics, and since 2012, GII.4 Sydney has been the dominant variant^2,3^. The major capsid protein VP1 is central to infectivity and immune recognition^4^, and VP1-based virus-like particles (VLPs) are considered promising vaccine candidates^5–9^. Yet, how the icosahedral HuNoV capsid responds to antibody pressure at the atomic level is still unclear. Here, we present the first atomic structure of GII.4 Sydney capsid determined by cryo-EM, revealing the structural features that likely contribute to its sustained dominance. Using focused refinement^10,11^, we uncovered the remarkable capsid plasticity in response to symmetry-breaking binding of neutralizing and non-neutralizing antibodies. VP1 adopts 11 distinct conformational modalities to accommodate asymmetric antibody binding, and antibody-disrupted VLPs reassemble into smaller particles with five additional VP1 states. This previously unrecognized extent of conformational adaptability provides key insights for designing next-generation VLP- or mRNA-based vaccines targeting HuNoV infections.

## Introduction

In the arms race between viruses and host immunity, viral capsids must balance between evading the immune system and maintaining structural fitness. Capsid plasticity modulates receptor interactions and cellular entry, while sheltering vulnerable regions from immune targeting. Our understanding of antigen-antibody interactions in viruses at the atomic level largely comes from studies of antigen-binding fragments (Fabs) in complex with isolated antigens, with only a few examples involving intact capsids^12–15^. High-resolution structural analysis, either by X-ray crystallography or single-particle cryo-electron microscopy (cryo-EM), is straightforward if antibody binding preserves viral symmetry. However, when Fab binding disrupts the underlying capsid symmetry, standard reconstruction procedures with symmetry enforcement average the signal, yielding uninterpretable structures and making it difficult to visualize capsid conformational changes. Asymmetric reconstructions and focused refinement can overcome this limitation, as demonstrated for adeno-associated virus (AAV) and parvovirus capsids bound to neutralizing Fabs^12–15^. Yet, the extent to which a virus capsid can undergo conformational alterations to accommodate symmetry-breaking antibody binding, particularly those recognizing cryptic antigenic sites, remains poorly characterized. A detailed understanding of capsid malleability and conformational modalities is critical for designing vaccine strategies for rapidly evolving viruses with pandemic potential, such as GII.4 human noroviruses.

Following the development of a rotavirus vaccine, human noroviruses (HuNoVs) have become the principal cause of acute gastroenteritis worldwide^1^, with an estimated annual disease burden of 677 million cases^16^ and societal costs of $60.3 billion^17^. The hypervariability of the major capsid protein VP1 is responsible for the periodic emergence of new pandemic strains every 2–3 years that escape herd immunity through epochal evolution^18^ and poses the main challenge for the development of effective vaccines. Based on the VP1 sequence, NoVs are classified into 10 distinct genogroups (GI-GX) and at least 48 genotypes, with a single strain, GII.4 Sydney, from the GII.4 genotype, resisting epochal evolution and causing up to 80% of infections between 2012–2023^2,3^. Other genotypes, including GII.3 and GII.17, cause sporadic outbreaks in certain geographic locations and can temporarily overtake GII.4 Sydney as the primary cause of HuNoV infections^19–22^.

The study of HuNoV infectivity is further hindered by a lack of a sustainable virus cultivation system, with the field relying on VLPs produced by expression of the major capsid protein VP1 alone or with the minor capsid protein VP2 for structural and functional studies^1^. Recombinant (co-) expression of VP1 and VP2 produces mixed VLP populations, including the native-like T=3 icosahedral assembly, but also larger T=4 and smaller T=1 icosahedral particles^23–26^. VP1 comprises a shell-forming (S) domain connected to a protruding (P) domain by a flexible hinge (**Fig. S1**)^4,26^, which enables structural rearrangements in VP1 between resting/closed and raised/open conformations, with the P-domain nestled atop the shell or lifted and twisted above it, respectively^25–28^. P-domains include a conserved P1 region near the shell and a distal hypervariable P2 region that binds the initial host cell attachment factors, the histo-blood group antigens (HGBAs)^29–33^. Mutations in P2 aid evasion of neutralizing immune responses from HBGA-blocking antibodies and modulate HBGA preference^34–36^. Meanwhile, potential cross-reactive epitopes in the conserved P1 region are occluded in the resting capsid conformation^37–40^. Structural studies across different genogroups, including murine NoVs^27,28,41,42^, GII.3^42^ and GII.4 (HOV variant)^26^, have shown that this “viral breathing” from a resting to a raised conformation is influenced by pH, metal ions or bile. The only high-resolution structure of a native GII.4 capsid revealed that metal ions are critical for stabilizing the resting conformation in GII.4 HOV^26^, but it remains unclear if ion binding occurs in other strains, particularly the pandemic GII.4 Sydney.

The HuNoV conformational plasticity has important implications for antigenicity, with a VLP-based vaccine candidate generating mixed immune responses, including (1) broadly reactive but non-neutralizing or (2) neutralizing but strain-specific antibodies^40^. The former category likely arose from the presentation of cryptic P1 epitopes, which require VP1 to transition to a raised state, as suggested by X-ray crystallographic studies of the P-domain in complex with neutralizing antibodies^38,43^. In vaccines against enveloped viruses, detailed knowledge of antigen (spike protein) flexibility/stability has informed development strategies to stabilize antigens in the prefusion state, thereby eliciting efficacious immune responses^44–46^. Currently, the limited understanding of the magnitude of tolerated VLP plasticity relative to a natural virus, and of how HuNoV capsids accommodate antibodies with cryptic epitopes, is a bottleneck in designing effective VLP-based vaccine strategies that push the immune response towards functional neutralizing antibodies.

Here, we present the first atomic structures of the pandemic GII.4 Sydney capsid. The VLPs assemble into T=3 and T=4 particles, found in the resting and raised conformations, respectively. The native T=3 capsid exhibits greater conformational stability than earlier epidemic strains. It appears to have evolved the ability to stabilize the resting conformation independently of external factors by incorporating two amino acid substitutions. Using asymmetric reconstructions and focused refinement procedures, we reveal the remarkable structural malleability of the capsids in response to binding of a human-derived neutralizing Fab, NORO-320, and a non-neutralizing llama-derived single domain nanobody (VHH) M6. We identify eleven distinct VP1 conformations in the capsid to accommodate antibodies binding to conserved cryptic epitopes in the P1 region. VLPs disrupted by antibody binding reassemble into smaller T=1 particles, presenting five additional VP1 conformations. Overall, our study highlights the magnitude of conformational plasticity in a viral capsid and provides a framework for the rational design of more effective HuNoV vaccines.

## Results

### VLPs of pandemic GII.4 Sydney assemble into T=3 and T=4 icosahedral structures.

To perform cryo-EM analysis of GII.4 Sydney VLPs, we co-expressed the structural proteins VP1 and VP2 in Sf9 insect cells and purified the VLPs. Following vitrification, we collected 3,290 movies and processed the data using cryoSPARC, yielding over 30,000 particles for 3D reconstruction. The sample contained roughly equal numbers of particles with T=3 (13,400) and T=4 (16,900) icosahedral symmetry (**Fig. S2A**).

### Atomic structure of T=3 GII.4 Sydney VLP shows VP1 in the resting conformation lacking metal ions

After homogeneous refinement enforcing icosahedral symmetry, the T=3 VLP map achieved a maximum resolution of 2.84 Å and a low-resolution limit of 4 Å at the distal end of P2 (**Fig. S2B**), allowing us to unambiguously fit the 3 quasi-equivalent VP1 conformations (A, B, and C) of the icosahedral asymmetric unit into the density. The VLP map displayed an inner diameter of 230 Å and an outer diameter of 410 Å, consistent with the dimensions of the previously characterized T=3 VLPs from GI.1^4,25^, GI.7^25^, GII.2^25^, and GII.4 HOV^26^, as well as the native virions^47^ (Fig. 1A).

The T=3 VLP structure as expected contained 180 VP1 molecules, with 60 copies of chains A surrounding the 12 five-fold icosahedral hollows, and 60 copies each of chains B and C surrounding the 20 three-fold hollows (Fig. 1A). These quasi-equivalent VP1 states formed either “bent” A-B or “flat” C-C dimers based on the angles between shell monomers (Fig. 1B). The cryo-EM density allowed partial fitting of the N-terminal arms (NTAs) from residues N24 (A and C) and D12 (B), which formed a similar interaction network as in the GI.1^4^ and GII.4 HOV^26^ T=3 structures. We noted the poorer map quality around the C-C dimers, consistent with this region’s conformational flexibility observed in the GII.4 HOV crystal structure^26^. To improve the map resolution, we performed focused 3D classification over a C-C dimer extracted from a stack of symmetry-expanded particle images, followed by local refinement. With this approach, we were able to improve the resolution to 2.8 Å and identify 2 major C-C dimer conformations, distinguished by a 14 rotation of the P-domains relative to the shell (**Figs. S2C-D, Supplementary movie 1**). However, the overall Sydney chains superimpose onto the HOV structure (PDB: 7MRY), with an r.m.s.d. of <0.7 Å. However, a closer inspection of the region corresponding to the metal binding pocket in GII.4 HOV showed no significant density attributable to ion binding (Fig. 1C). This region is located at the base of P1 and adjacent to the hinge (**Fig. S2E**). Sequence comparison between the Sydney and HOV strains revealed mutation of two of the four ion-coordinating residues, L459Q and H460Y, but no variation in the hinge across historic and current GII.4 variants.

We investigated the effects of environmental factors, including pH and metal ions, on the Sydney capsid conformation using dynamic light scattering (DLS) to measure the hydrodynamic diameter of particles in solution. Since the cryo-EM model lacked the metal ion shown to stabilize the resting HOV VLP conformation^26^, we considered the possibility of ion loss during VLP purification and therefore supplemented with an excess of divalent cations. However, neither Mg^2+^ nor Ca^2+^ affected the average VLP diameter by DLS (Fig. 1D, **S2F**). Next, we tested the influence of buffer pH and EDTA-mediated chelation of any bound metals on the VLP conformation, but again observed no difference in particle diameter (Fig. 1E, **S2F**). This apparent rigidity contrasts with the conformational changes of GII.4 HOV and murine NoV mediated by pH, metal ions, and bile acid, and it is possible that the Sydney VP1 transitions between resting and raised states only partially or too slowly to measure by DLS.

### T=4 structure shows VP1 in the raised conformation

The T=4 VLP map achieved a lower resolution of 3.89 Å after homogeneous refinement and a broader resolution range up to 6.9 Å at the distal end of P2 (**Fig. S3A**). Despite this, we were able to fit the shells of quasi-equivalent A, B, C, and D chains into the density and to reconstruct the rest of VP1 by placing the P-domains from the T=3 structure and manually building the connecting hinge into the density map in Coot (Fig. 1F and **S3B**). The T=4 structure measures 300 Å inner and 490 Å outer diameters and comprises 60 A chains around 12 five-fold hollows and 60 copies each of chains B, C and D forming 30 six-fold hollows (Fig. 1F). Overall, the chains are superimposable with the T=4 GII.4 Minerva structure (PDB: 6OUU), with an r.m.s.d. of 1.3 Å. The N-terminal 46 residues of the NTA in our structure, as in Minerva, are disordered, suggesting that inter-shell interactions are sufficient to support the T=4 scaffold with minimal or no input from the NTAs (residues 1–46), in agreement with the report that truncation of the NTA (residues 1–20) sustains VLP assembly^48^.

The shells of quasi-equivalent A-B and C-D dimers form ~10° wider angles than those in the T=3 VLP, consistent with the larger curvature of T=4 particles (Fig. 1G, **S3C**). Additionally, the C-D dimer P-domains tilt and twist closer to the shell of chain C by 35 Å and 28° compared to the A-B conformation. Because of the lifting and twisting of P-domains relative to the shell in the raised state, no true two-fold symmetry axis exists in T=4 VLPs.

Although the mechanism by which VP1 transitions between resting and raised states is unclear for GII.4 Sydney, we compared the hinges of quasi-equivalent chains from our T=3 and T=4 models to identify the structural features that enable this motion. After superimposing all VP1 chains at the shell, we observed that the hinges adopt a similar conformation up to residue 218, beyond which the T=3 hinges turn and continue horizontally, whereas the T=4 hinges splay upwards (**Fig. S3D**).

### Broadly cross-reactive M6 nanobody binds to a cryptic epitope at the S-P interface

To study how single-domain antibodies bind to the HuNoV capsid and modulate its plasticity, we selected M6 from a panel of llama-derived nanobodies produced after animal immunization with a GII.4 VLP strain from 2004^49^. We chose M6 due to its broad reactivity against GII.1, GII.2, GII.3, GII.4, GII.6, GII.7, and GII.14 VLPs and non-overlapping epitopes with other nanobodies from the panel^49^. The M6 epitope was determined from the crystal structure of the nanobody bound to a P-dimer at 2.2 Å resolution (Fig. 2A). Only crystals containing M6 bound to GII.3 P-domain, but not GII.4 Sydney, produced good diffraction data used for structural determination.

In the crystal structure, each P-domain interacts with a single M6 molecule with a buried surface area of 600 Å^2^. The nanobody recognizes an epitope on the P-domain near the shell interface and distal to the HBGA binding pocket (**Fig. S4B**). Predictably, there is no conformational change between the M6-bound P-dimer and the HBGA-P-dimer complex, with an r.m.s.d. of 0.4 Å (**Fig. S4C**). The epitope recognized by M6 is a linear stretch of amino acids on the P-domain located between residues E526-T534, with minor contributions from four additional residues (F434, P435, L482, R484), an unusual aspect, since nanobodies commonly recognize conformational epitopes^50^. Three of the residues in the linear sequence, E526, N530, and T534, as well as R484, are involved in hydrogen bonding with M6, while the rest make van der Waals contacts with the complementarity-determining regions (CDRs) (Fig. 2B, **S4A**). The peptide backbone of T534 binds to the sidechain of N32 through two H-bonds. E526 is recognized by the sidechain of R110 and the peptide amino group of V109, which form H-bonds to the same oxygen atom of the sidechain carboxyl group. N530 is stabilized by three H-bonds between its sidechain and residues N96, N98, and G33, and one H-bond with N98 between their respective peptide amino and carboxyl groups. Lastly, the sidechain of R484 binds to the peptide carboxyl group of D107.

Sequence alignment of the M6 epitope identified in our crystal structure reveals 56–100% identity across 26 GII genotypes, and 80% identity between GII.3 and GII.4 Sydney sequences (**Fig. S4D**). The high degree of conservation of the linear epitope allows us to use the crystal structure in analyzing the binding mode of M6 to Sydney VLPs.

### M6 fails to neutralize HuNoV infection in HIEs

The crystal structure clearly showed that M6 binds to an inaccessible location on the P-domain, hidden at the P1-S interface in the resting VP1 conformation. Other nanobodies and Fabs with occluded epitopes on P1 show variable breadth of HuNoV neutralization^32–35,38,47^; therefore, we tested the effects of M6 on viral infectivity of the GII.3 and GII.4 Sydney genotypes in human intestinal enteroids (HIEs) as the replication system^52^. We measured viral genome equivalents at 24 hr post-infection from stool filtrates, mixed with varying concentrations of M6 relative to an untreated control. Both genotypes replicated similarly to the control condition, even at the highest nanobody concentration (Fig. 2C, D). In parallel, we tested nanobody M5 as a positive control, which neutralizes GII.4 Sydney infection in HIEs with a 90% inhibitory concentration (IC90) of 82 ng/ml (**Fig. S4F, G**). It appears that M6 does not affect viral fitness or integrity, nor does it prevent interactions between the capsid and/or (co-)receptors, presenting an opportunity to study the extent of VP1 conformational flexibility tolerated to accommodate a non-neutralizing nanobody.

### M6 binding alters the GII.4 VLP conformation and integrity

The GII.3 and GII.4 Sydney VLPs responded to addition of M6 with a 3–5 nm reduction in the average hydrodynamic diameter and a global downshift in measured radii in DLS assays (Fig. 2E, **S4F-G**). This change is considerably less than the ~20 nm reduction in average diameter of both GII.3 and GII.4 VLPs by the neutralizing and destabilizing nanobody M4^38^. To better understand how M6 affects the VLPs, we imaged GII.4 Sydney particles after overnight incubation with M6 using high-resolution cryo-EM. While the untreated sample contained an equal mix of T=3 and T=4 particles, addition of M6 completely destabilized the T=4 VLPs, giving rise to smaller, ~30 nm T=1 particles in a 2:1 ratio over the T=3 VLPs (Fig. 2F, Tables 1, 3). As shown, the original sample lacked T=1 VLPs, and the disappearance of T=4 VLPs after nanobody treatment suggests that VP1 dimers from disrupted T=4 VLPs reassemble into smaller T=1 particles.

### T=3 VP1 dimers exhibit 6 different conformations to accommodate binding of M6

Binding of M6 to T=3 particles caused motion in the P-domains, leading to blurring of the density (**Fig. S5A**). The T=3 VLP reconstructions resulting from particles incubated with M6 exhibited the same inner diameter as untreated particles (250 Å) and a 40 Å larger outer diameter, caused by lifting of P-domains above the shell (Fig. 2F, **S5B**). As a result of M6 binding, the VLPs no longer maintained strict icosahedral symmetry, and reconstruction of whole particles yielded incomplete, choppy densities around the P-domains. To overcome this, we symmetry-expanded the stack of particle images using the I point group. Then, we subtracted the VLP density using a mask over an A-B or C-C dimer to generate new stacks of particles containing only the icosahedrally-related A-B or C-C dimers. We employed 3D classification of the new particle stacks to separate distinct VP1 dimer conformations, followed by a local refinement step that generated the final models used in structure building (**Fig. S5D-E**).

### A-B dimers bind M6 with three modalities

We identified 3 distinct classes of A-B dimers bound to M6, all with good definition (**Fig. S5D**). The first two classes displayed broad resolution ranges of 3.6–7.5 Å and 2–5.5 Å, respectively, with most of the variability clustered in the P-dimer and M6 regions, while the third class exhibited a narrower resolution range of 3–4.3 Å.

Across all structures, the P-domains are raised above the shell to reveal the M6 epitope. In the first class, the P-dimer lifts by 16 Å off the shell and rotates by ~38° about the local 2-fold axis to accommodate two M6 molecules (Fig. 3B, Table 5). The nanobody binding mode in the cryo-EM map is identical to that in the crystal structure. The other two classes comprise a single M6 chain bound to the P-dimer, either to the A chain (from the 5-fold axis hollow) or to the B chain (from the 3-fold axis hollow, Fig. 3A, B). The raised P-dimers in these classes twist toward one of the shell domains by 34° and 24°, respectively, to fit the single M6 chain. Apart from the 34° rotation, the dimer bound from the A chain also moves by 11 Å (Table 5). In the single M6-bound classes, the P-domain of the unbound chain contacts its own shell domain, thus completely covering the other M6 epitope. The conformation of A-B dimers with M6 binding from the 3-fold hollows contains 7 times more particles than the other two classes (Table 6).

Despite the P-dimer motion, the shell curvature remains unaffected by M6 binding (**Fig. S5C**). The flatter curvature of the T=4 particle shell may restrict the swing of the P-domains necessary to accommodate the nanobody without steric clashes. By superimposing the flatter shell dimer from a T=4 A-B dimer onto an M6-bound T=3 dimer, we observe clashes between the unbound P and its corresponding S domain (Fig. 3C). The T=3 and T=4 shell dimers are not perfectly superimposable due to a 10° curvature difference between the monomers. To analyze possible clashes caused by M6, we aligned the two shell dimers at the monomer interface (residues 85–100) for the best approximation; however, we recognize the limitations of this modeling.

### C-C dimers bind M6 with three modalities

Next, we analyzed the C-C dimers bound to M6. These also fell into three categories, with a 2.8 Å resolution around the shell and a range between 3.3–7 Å in the P-domain and nanobody regions (**Fig. S4E**). In all three categories, the P-domains are raised above the shell and bound to either two M6 molecules (largest class) or to a single M6 from either one of the C chains at the 3-fold axes (Fig. 4A, B). The double-bound C-C dimer conformation contains nine times more particles than the single-bound classes (Table 6). To accommodate both M6 molecules, the double-bound C-C dimer lifts by ~18 Å off the shell and rotates by 40° about the icosahedral 2-fold axis (Fig. 4B, Table 5). In both single-M6 classes, the P-domains lift by 5–9 Å, with the M6-bound chain raising more than the unbound one to fit the nanobody. The raising motion is accompanied by a 13–16° rotation about the icosahedral 2-fold axis (Table 5). The shells of M6-bound C-C dimers remain stable and are superimposable onto the native T=3 C-C shells (**Fig. S5C**). Unlike the single M6-bound A-B dimers, the P-domains of C-C dimers do not touch either shell domain, despite the flatter shell conformation (Fig. 3B and 4B). See **Supplementary movie 2** showing various M6-bound VP1 conformations.

Modeling of the T=4 C-D shells onto any M6-bound C-C dimer reveals steric clashes between the nanobody and the shell. Again, we aligned the shells of T=3 and T=4 C-C/C-D dimers via the monomeric interface (residues 85–100). Thus, it is possible that the wider T=4 shell creates steric clashes upon M6 binding, leading to T=4 particle disassembly (Fig. 4C).

### Broken T=4 particles reassemble into T=1 VLPs displaying additional VP1 conformational states

Treatment of GII.4 Sydney VLPs with M6 nanobody destabilized the T=4 particles, leading to some VP1 dimers reassembling into T=1 VLPs (**Fig. S6A**). These particles comprise 60 A chains arranged into 30 A-A dimers around twelve 5-fold axes and twenty 3-fold axes (Fig. 5A). The whole-particle reconstruction generated a 2.8 Å map around the T=1 shell, with lower resolution around the blurred P-domains (**Fig. S6B**). Using the same processing workflow as before, we identified three distinct classes of A-A dimers bound to either one or two M6 molecules. The single-bound classes displayed higher resolution, up to 4.5 Å, while the double-bound class exhibited a broader resolution range, up to 6 Å, and poorer definition of one of the M6 chains (**Fig. S6C**). To accommodate two nanobodies, the A-A dimers undergo a lift of 14 Å and a 32° rotation, recapitulating the motion of double-bound A-B and C-C dimers seen in T=3 VLPs (Fig. 5B, Table 5). In both single-bound nanobody classes, the P-dimers rotated by 25 towards the shell of the unbound chain, creating a contact between P and S, akin to the motion of the A-B dimer with M6 bound from the 3-fold axis in T=3 VLPs (Fig. 5B, Table 5).

### Crosstalk between VP1 dimers generates mutually exclusive conformational states

T=3 VLPs consist of 60 A-B dimers and 30 C-C dimers, each with two possible epitopes for M6. However, we discovered A-B and C-C dimer conformations with a single M6 position occupied, indicating that the full set of 180 epitopes remains unsaturated by the nanobody due to steric clashes that prevent access to certain sites. We sought to understand the crosstalk between adjacent VP1 dimers and the factors that dictate the choice between the single- and double-bound conformations. To that end, we selected one asymmetric unit on the VLP containing an A, B and C chain, and extracted the 3 dimers formed by those chains from the symmetry-expanded particle stack (Fig. 6A, B). Next, 3D classification revealed the combinations of dimer conformations present in the dataset.

Most particles (~71%) contained a double-bound C-C dimer, and the A-B dimers bound via the B chains (class B-CC-B, Fig. 6C), consistent with the most abundant conformation for each of A-B and C-C dimers (Table 6). Two other classes also contained double-bound C-C dimers, but only one of the A-B dimers bound via chain B, with the other bound via A (classes B-CC-A and A-CC-B). The last 2 classes contained single-bound C-C dimers and one of the A-B dimers in a double-bound form (B-C_2_-AB and AB-C_1_-A).

We did not detect classes with both A-B dimers bound via A chains (from the 5-fold side), with both A-B dimers in double form, or with more than one double-bound dimer. Having both A-B dimers in a double-bound form causes steric clashes at the center of the asymmetric unit, but having one double-bound C-C and one double-bound A-B is theoretically permitted. Similarly, having both A-B dimers bound from the 5-fold axis does not create any clashes.

### Broadly neutralizing Fab NORO-320 expands VP1 binding modalities in T=4 VLPs

Thus far, our studies have focused on the GII.4 capsid plasticity in response to a single-domain non-neutralizing nanobody. Next, we wanted to uncover the effects of a neutralizing human Fab on the capsid integrity using a similar cryo-EM processing workflow. We previously reported that IgA-derived Fab NORO-320 exhibits broad reactivity against multiple GII genotypes (GII.3, GII.4, GII.6, GII.13, GII.17) and neutralizes GII.4 Sydney infection in both IgA and Fab forms, independently of the HBGA site^38,53^. NORO-320 binds to a distinct cryptic epitope in the P1 subregion than M6 (**Fig. S7A**), which becomes available for binding from the icosahedral hollows in the raised VP1 conformation (**Fig. S7B**). 2D classification of GII.4 Sydney particles incubated with Fab NORO-320, in a striking contrast to M6 binding, revealed the disappearance of the native T=3 form and its replacement with smaller T=1 VLPs. The T=4 particles, however, retained the original dimensions, but produced blurred P-domain densities (**Fig. S7C**).

NORO-320 treatment destroyed ~80% of the particles in the original sample, decreasing the maximum achieved resolutions to 4.7 Å in the shell region. We applied the same protocol to resolve symmetry-breaking features and identified Fab densities at the A-B, C-D (T=4) and A-A (T=1) dimers (**Fig. S7**). Although lacking atomic detail, the cryo-EM maps allowed us to fit the shell and P-dimers from our T=4 or T=1 reconstructions and Fab NORO-320 from the crystal structure (PDB: 7JIE).

From 3D classification, A-B dimers were either unbound (68.1%), with NORO-320 bound from the 5-fold hollow (14.5%) or the quasi 6-fold hollow (17.4%, Table 6). Regardless of the Fab binding direction, the A-B dimers did not present any significant conformational changes compared to unbound dimers from the T=4 structure (**Fig. S8A**). Separately, C-D dimers fell into 4 classes: unbound (41.3%), with Fab bound from the C shell direction (left hand side, 12%) or D shell direction (right hand side, 19.5%) and double-bound (27.2%, Table 6). Again, all Fab-bound dimers were structurally similar as C-D dimers from the T=4 model, revealing that NORO-320 binds directly from the icosahedral T=4 hollows without imposing structural constraints (**Fig. S8B**). All dimers composing the T=1 particles were either flexible and presumed unbound (~63%) or bound by two Fabs simultaneously (**Fig. S8C**), with the P-domains twisting toward either shell domain and assuming almost identical conformations to the single M6-bound A-A dimers from the T=1 VLPs (Figs. 5B, **S8C**), with only a 7° rotation between the respective structures. See **Supplementary movie 2**, which lists various Fab-bound VP1 conformations.

To understand the crosstalk between unbound and Fab-bound A-B and C-D dimers that leads to complete occupation of the T=4 icosahedral hollows by NORO-320, we repeated the particle subtraction step on the symmetry-expanded particle stack with cylindrical masks covering the VP1 dimers around a 5- or quasi 6-fold hollow (Fig. 7A). Subsequently, we performed 3D Classification using narrower cylindrical focus masks that covered only the hollows.

This revealed that only one NORO-320 molecule covers the 5-fold hollows, with an equal likelihood of binding to any of the five VP1 dimers (Fig. 7B). On the other hand, the larger quasi 6-fold hollows accommodate two NORO-320 molecules, grouped into four separate classes. In almost half of the quasi 6-fold hollows, NORO-320 was bound to diametrically opposite C-D dimers from the D chain shell side (class 1–4, Fig. 7C). The second most populous class again contained the Fabs bound to opposite C-D dimers, but from the C chain shell side (class 6–3, ~24%). In the last two classes, the remaining quasi 6-fold hollows contain one Fab bound to one of the A-B dimers and another Fab bound to a C-D dimer (classes 1–5 and 4–2). It is unclear why NORO-320 does not simultaneously bind the diametrically opposite A-B dimers at the quasi 6-fold axes. Additionally, the disruption of T=3 particles is also unclear, since the epitopes for NORO-320 are accessible in the raised capsid conformation. However, this state is likely a minor conformation of GII.4 Sydney VP1 dimers within the T=3 VLP. As a result of stochastic VP1 raising and P-domain flexibility, our hypothesis is that multiple dimers at the icosahedral hollows may become bound by NORO-320, causing steric clashes between Fabs that result in the destabilization of T=3 VLPs.

## Discussion

In this study, we present the first atomic structures of the pandemic GII.4 Sydney VLP capsids in both their native T=3 and non-native T=4 conformations determined using high-resolution cryo-EM. These structures provide insights into how evolutionary changes in capsid stability and flexibility contribute to the global predominance of this variant. We also report cryo-EM analyses using focused refinement techniques to probe capsid plasticity upon binding to a broadly cross-reactive non-neutralizing and a neutralizing antibody, providing structural insight into how capsid adaptability contributes to immune evasion. Although previous work on AAV and parvoviruses demonstrated that antibody engagement can distort capsid symmetry, our findings show that HuNoV VP1 samples a far broader range of conformational states than previously documented for any icosahedral virus^14,15^. The ability of the GII.4 Sydney capsids to accommodate symmetry-breaking antibodies without compromising the overall structural integrity indicate a finely tuned balance between stability and flexibility that likely contributes to their epidemiological predominance.

Apart from the atomic structure of the T=3 capsid of the GII.4 Sydney 2012 variant reported here, the only other GII.4 structure with native-like T=3 symmetry is that of the 2002 variant (HOV)^26^. Although the modular domain organization is well conserved between the two, a major distinction is the absence of ion density in the GII.4 Sydney structure. Of the four residues that coordinate ion binding in the 2002 variant, only two are conserved in the Sydney 2012 variant. The most striking change is the substitution of a histidine residue (H460) in the 2002 variant that sandwiches the ion with its protonatable imidazole ring between the opposing subunits at the dimeric interface with a tyrosine in the 2012 variant (Fig. 1C), which alters local electropositivity and contributes to the loss of ion binding^54^.

Interestingly, comparison of VP1 sequences across all GII.4 variants reveals strong conservation of this histidine residue in pre-2007 GII.4 variants, which is consistently altered to tyrosine or to less electropositive, non-protonatable residues such as phenylalanine in post-2007 variants, including GII.4 Sydney (**Fig. S9**). This shift likely indicates differences in capsid stabilization strategies, with post-2007 variants potentially reflecting evolutionary drift favoring structural adaptability or flexibility to enable altered presentation of antigenic sites. Consistent with this notion of differential capsid stability is our observation that the size of the GII.4 Sydney VLPs, despite being a mixture of 40-nm T=3 and 50-nm T=4 particles, remain unchanged under varying pH conditions or treatment with EDTA, unlike the behavior observed for the GII.4 2002 variant^26^. One possibility is that such stability, without reliance on the external environment such as pH and ionic strength, may limit exposure of conserved P1 epitopes during natural infection, thereby limiting the immune system’s ability to generate broadly neutralizing responses and contributing to the increased pandemic potential of the post-2007 GII.4 variants.

Another important observation regarding GII.4 VLPs, one that may have implications for VLP-based vaccine design strategies (discussed further below), is their apparent ability to assemble into capsids with both T=3 and T=4 symmetries (sometimes, including T=1)^23–25^. The resting conformation of VP1 appears favored in the T=3 GII.4 capsid (HOV and Sydney), regardless of ion stabilization. In contrast, both the available cryo-EM structures of T=4 GII.4 capsids (Minerva^25^ and Sydney variants) show VP1 in the raised conformation. It is unclear why the raised conformation is preferred in the T=4 particles. One possibility is that the greater curvature required for T=4 particle formation can occur only when VP1 adopts the raised state, potentially facilitated by the altered dynamics of the flexible hinge. It is important to emphasize that although VLPs self-assemble into mixed T=3 and T=4 populations, this duality is not present in native virions^52,55^. All native calicivirus structures determined to date—including feline calicivirus^11,56^, San Miguel sea lion virus^57^, rabbit hemorrhagic disease virus^58,59^, and murine norovirus^28,42^—exhibit T=3 symmetry. This likely reflects fundamental differences in the initiation of capsid assembly between VLPs and virions, in which the combined presence of the genome, VPg, and VP2 helps enforce the native T=3 architecture.

Our key discovery of the unprecedented capsid malleability that accommodates symmetry-breaking binding by both neutralizing and non-neutralizing antibodies illustrates the dynamic nature of HuNoV antigenicity. The binding of M6 and NORO-320-Fab stabilizes VP1 rearrangements, revealing cryptic epitopes in the P1 subdomain that are masked in the resting conformation and facilitating their binding in various modalities within the confines of icosahedral assembly, maintained by S-domain interactions. These findings provide a possible mechanistic explanation for why VLP-based vaccines often elicit antibodies targeting non-neutralizing epitopes^40,60^. The mixed T=3 and T=4 particles, as well as disrupted particles, each presenting distinct antigenic landscapes, could bias the immune response toward epitopes rarely accessible on infectious virions. Another finding that could further complicate the antigenic landscape for VLP-vaccine design is the disassembly of T=3 or T=4 assemblies upon antibody binding, followed by their reassembly into smaller antibody-bound (pseudo) T=1 particles, revealing additional conformational states of VP1. Our studies here provide a roadmap for the design of next-generation immunogens that elicit potent neutralizing antibodies that block HBGA binding or stabilize the resting conformation, while minimizing the presentation of cryptic P1 epitopes^34–36^.

Our studies also raise an important question regarding the role of capsid dynamics in human norovirus infection. Viral breathing has been implicated in receptor engagement, uncoating, and environmental resilience in other non-enveloped viruses^61,62^. In the case of human noroviruses, there has to be a fine balance between capsid stability and flexibility. Subtle conformational flexibility may be essential for transiently sampling the raised conformations to optimize interactions with HBGA, cellular receptors, or co-receptors for successful infection. However, excessive flexibility may expose unwanted regions in the capsid to the immune system. Although the cellular receptors for HuNoVs are currently unknown, this notion is supported by studies with murine norovirus (MNV), which relies on engaging the CD300lf receptor in the gut epithelium, with the VP1 resting conformation stabilized by low pH and high concentrations of metal ions and bile in the intestinal milieu^27,42,63,64^. In the resting conformation, MNV exhibits enhanced receptor binding and reduced antibody accessibility. It is proposed that viral transit into the lymphatic system with neutral pH, low bile, and low metal ions reverts virions to a raised conformational state, with reduced affinity for the receptor, exposing the conserved P1 epitopes to immune surveillance only outside the gut. This mechanism of viral shapeshifting is suggested in viruses such as MNV, SARS-CoV-2, and HIV, as a strategy to dilute the neutralizing immune response by eliciting antibodies against cryptic epitopes that are not functional at the site of infection^28,65–68^.

In summary, our study reveals novel insights into how evolutionary changes could contribute to differential capsid stability and antigenicity, as well as to the predominance of post-2007 variants. Second, by uncovering the previously unappreciated breadth of VP1 conformational states (**Supplementary movie 2**) and their modulation upon antibody binding, this study highlights that precise control of capsid architecture is a critical factor in designing VLP-based vaccine strategies that can elicit broadly protective, functionally neutralizing antibodies. Such an approach is analogous to prefusion stabilization strategies used in respiratory virus vaccines^44–46^. Beyond establishing a strong foundation for rational antigen engineering, our findings open new avenues for understanding how capsid plasticity in a virus shapes viral evolution and immune escape.

## Materials and methods

### Expression and purification of GII.4 Sydney and GII.3 VLPs

VLPs were produced in a baculovirus expression system using open reading frame 2 (ORF2) + ORF3+ untranslated region (UTR) sequences for different HuNoV strains. The GII.4 [P31] Syndey HuNoV (Sydney/NSW0514/2012/AU, GenBank #JX459908.1) and GII.3 [P21] HuNoV (Hu/TCH04–577/USA, GenBank #AB365435.1) VLPs were expressed from recombinant baculoviruses (pVL1393 and PVL1392 for the GII.4 and GII.3 strains, respectively) in Sf9 cells infected with a MOI of 5 for 10 days in the presence of protease inhibitors, leupeptin, pepstatin A, aprotinin, and PMSF, as previously described^69,70^. VLPs were purified from clarified Sf9 lysates by centrifugation of the supernatant over a 30% sucrose cushion, before centrifugation at 120,000x *g* for 3 hrs at 4°C. The resulting pellet was soaked in 200 μl PBS per tube overnight at 4°C. Resuspended VLPs were mixed with PBS + CsCl (1.28 mg/ml) in a 10 ml total volume and ultracentrifuged at 150,0000x *g* for 18 hrs at 4°C. The opaque band corresponding to intact particles was collected with a micropipette, and the VLPs were dialyzed in PBS pH 6 overnight at 4°C.

### Expression and purification of GII.3 P-domain, M6 and Fab NORO-320

The GII.3 (TCH04–577) P-domain was cloned into a pMal-C2E vector (New England Biolabs) with an N-terminal 6xHis-MBP tag separated by a tobacco etch virus (TEV) cleavage site. The protein was expressed in BL21 (DE3) cells overnight at 18°C after induction with 0.2 mM IPTG. The protein was captured on Ni-NTA resin (QIAGEN) in cell lysis buffer (CLB: 20 mM Tris, 200 mM NaCl, 10 mM imidazole, 2.5 mM MgCl_2_, 0.007 % BME, pH 7.4), washed with CLB + 30 mM imidazole, and eluted with CLB + 300 mM imidazole. The 6xHis-MBP tag was cleaved by TEV protease incubation (1:100 w/w) during overnight dialysis in CLB without BME. The tag was removed by negative-affinity chromatography of the cleaved protein stock using Ni-NTA and Amylose resin (New England Biolabs).

The M6 nanobody was cloned into a pHEN6 vector with an N-terminal PelB periplasmic export sequence and a C-terminal 6xHis tag. The protein was expressed in BL21 (DE3) cells overnight at 28 °C after induction with 1 mM IPTG. The cells were subjected to osmotic shock to extract the periplasmic nanobody by first resuspending the pellet in 12 ml TES (0.2 M Tris, 0.5 mM EDTA, 0.5 M sucrose, pH 8.0) per liter culture overnight at 4°C. Then, the cells were mixed with 5 mM MgSO_4_ (24 ml/ 1 l culture) for 2 hrs at 4°C and centrifuged at 11,000x *g* for 30 min at 4°C. The osmotic shock was repeated with another TES treatment for 1 hr followed by MgSO_4_ incubation for 2 hrs. The nanobody was captured on Ni-NTA resin (QIAGEN) and further purified using a Pierce Strong Cation Exchange column.

The Fab NORO-320 was produced as described previously^38^. Briefly, supernatant from CHO cells transiently expressing both mammalian codon-optimized heavy and light chain fragments was passed through a 0.45 μm filter, and the Fab was purified by affinity chromatography on a column containing KappaSelect resin (GE Healthcare Life Sciences).

### Cryo-EM sample preparation and data collection

GII.4 Syd VLPs or GII.4 Syd VLPs mixed with M6 / NORO-320 at 1:4 molar ratio were dialyzed into PBS pH 6.0 overnight. Aliquots (4 μl) were applied to graphene oxide-coated 1.2/1.3 holey carbon-coated copper grids (Quantifoil R1.2/1.3, 200 mesh, Cu) and incubated for 10 s. Grids were blotted for 2.5 s at 18°C, 100% humidity, and 0 blot force and plunge-frozen in liquid ethane using a Vitrobot (Thermo Fisher Scientific). Cryo-EM imaging was performed on a Titan Krios G3 TEM (Thermo Fisher Scientific) operating at 300 keV equipped with a post-column BioQuantum Energy Filter. Images were recorded with EPU using a K2 Summit direct detector (Gatan Inc.) at a nominal magnification of 130,000 for untreated and antibody-treated VLPs, the latter collected in super-resolution mode with pixel sizes of 1.08 Å and 0.54 Å, respectively. Antibody-treated particles were later resampled to 1.4 Å (T=3 and T=4 VLPs) and 1.08 Å (T=1 VLPs). Movies were collected with a defocus range of 0.5 to 2.5 μm, with a total accumulating electron dose of ~50 e^−^/Å^2^. The datasets were processed with CryosSPARC^10^ v4.6.0 (untreated VLPs) and v4.7.0 (antibody-treated VLPs). Each movie stack was split into 35 frames to allow beam-induced motion correction using the Patch Motion Correction. CTF parameters were determined with Patch CTF estimation, and ~100 VLPs corresponding to each of T=1, T=3 and T=4 forms were picked manually to generate templates for particle picking. Curated 2D classes of each VLP type were used in Ab-Initio reconstructions and Homogeneous Refinement with icosahedral (I) symmetry imposed. The gold standard Fourier Shell Correlation (GSFSC) with 0.143 cutoff was used for resolution determination.

To resolve symmetry-breaking features like the antibody-binding modes, the particle stacks were symmetry-expanded with I point group symmetry (**i**). The expanded stacks and 3D volumes from Homogeneous Refinement were recentered to a new position corresponding to the center of a VP1 P-dimer (A-B or C-C) using 3D xyz coordinates measured in UCSF ChimeraX-1.11.1^71^ (**ii**). The recentered volumes were then used to generate particle subtraction masks. Briefly, a cylindrical shape was placed over the VP1 dimer at the new center, converted into a density map, and resampled on the same grid size as the recentered volume in ChimeraX (**iii**). The resulting cylindrical map was converted into an inverted subtraction mask in CryoSPARC with 0 pix dilation radius and 8–10 pix soft padding and used in particle subtraction (**iv**) with the recentered particles and volume from step (**ii**). Different conformations were identified in the subtracted particles using 3D classification with a filter resolution of 12 Å (**v**). The same cylindrical map from (**iii**) was converted into a refinement mask with a 2-pixel dilation radius and 14-pixel soft padding and used in Local Refinement to improve the resolution of good 3D classes with pose and shift gaussian priors enabled and recentering rotations and shifts at each iteration.

### Cryo-EM structure determination and refinement

The structures of T=3 and T=4 GII.4 Sydney VLPs were reconstructed by fitting the asymmetric units of GII.4 HOV (T=3; PDB 7MRY) and GII.4 Minerva (T=4; PDB 6OUU) into the best Homogeneous Refinement maps from CryoSPARC using ChimeraX. Missing N-terminal residues and amino acid differences between those strains and GII.4 Sydney were modeled manually in Coot^72^, with iterative cycles of Real Space Refinement in PHENIX^73^ and further model building in Coot.

Structures of M6-bound VP1 dimers were built by fitting the A-B or C-C shell dimers from the T=3 model and the crystal structure of GII.3 P-domain with one or two M6 molecules into the Local Refinement maps in ChimeraX. GII.4 Sydney P-dimers were superimposed onto the crystal structure. The Fab NORO-320-bound structures were generated similarly, starting from A-B or C-C shell dimers from the T=4 model and fitting the crystal structure of GII.4 P-dimer with NORO-320 (PDB 7JIE) as rigid bodies. Flexible hinges were built manually in Coot. VP1 models were subjected to iterative cycles of Real Space Refinement and model building in PHENIX and Coot, while Fab and M6 chains were maintained with fixed occupancies and rotamer search was disabled for regions with resolution lower than 5Å. Refinement statistics are given in Tables 1, 3 and 4.

### Dynamic light scattering

VLPs were diluted in PBS pH 7.0 or as specified to 0.2 μM. Metal ions (20 μM), EDTA (3.2 μM) or M6 (0.8 μM) were added and incubated for 1 hr at 37°C. The VLP hydrodynamic diameters were measured with a ZetaSizer Nano instrument (Malvern Instruments) after equilibration at 25°C for 2 min. Three rounds of measurements were performed, with automatically determined duration and standard parameters (Refractive Index 1.330, Viscosity 0.888 cP, Temperature 25°C).

### Virus neutralization assays

The neutralizing activity of M6 against GII.4 Sydney (Sydney/NSW0514/2012/AU, GenBank #JX459908.1) and GII.3 (Hu/TCH04–577/USA, GenBank #AB365435.1) was measured in human intestinal enteroids (HIEs) as previously described^38,74,75^. In brief, J4/FUT2-KI HIEs were differentiated as monolayers in collagen IV-coated 96-well plates using OGMd medium (Stem Cell Technologies). Serial 5-fold dilutions of M6 nanobody were prepared in CMGF(−) medium supplemented with 500 μM sodium glycochenodeoxycholate (GCDCA) (Sigma, G0759), mixed 1:1 with 100 TCID50 GII.4 Sydney [P31] or GII.3 [P21], and preincubated for 1 hr at 37C. Nanobody-virus mixtures were inoculated in triplicate J4/FUT2-KI wells for 1 hr at 37°C; monolayers were washed twice with CMGF(−), then cultured in OGMd medium supplemented with 500 M GCDCA for 24 hrs. RNA was extracted at 1 hpi (input baseline) and 24 hpi using KingFisher Flex/MagMax Viral RNA kit; GEs quantified by RTqPCR. Percent GE reduction was calculated relative to medium control (100%).

### Crystallization of GII.3 P-domain and M6 complex

The protein complex was assembled from GII.3 P-domain and M6 mixed in a 1:1.5 molar ratio at 4°C for 1 hr and separated on a S200pg 16/60 size exclusion column in 20 mM Tris, 150 mM NaCl, pH 7.4. The peak corresponding to the complex was validated by SDS-PAGE to contain both proteins, and the peak fractions were pooled and concentrated to 9 mg/ml. Crystallization screens using the hanging-drop vapor diffusion method with commercially available kits were set up with a Mosquito nanoliter handling system (SPT LabTech) at a 1:1 protein: crystallization solution ratio. GII.3 P-domain-M6 crystals grew in 0.3 M succinic acid, 0.1 M citric acid·NaOH, pH 3.5, 22.5% PurePEGs cocktail, 0.01 M phenol, and harvested in 25% glycerol as cryoprotectant.

### Data collection and crystal structure determination

Diffraction data from GII.3 P-M6 crystals were collected on beamline 8.2.2 of the Advanced Light Source (Berkeley, CA) and processed with the XDS^76^ suite. The GII.3 P-dimer (PDB 6IS5) and M4 nanobody (PDB 8G0W) were used as search models for molecular replacement with PHASER^77^. Iterative cycles of manual model building in Coot and refinement in PHENIX were performed. Data collection and refinement statistics are given in Table 2. Model visualization was done in ChimeraX, and the interactions between GII.3 P-domain and M6 were analyzed in LigPLOT+ v.2.1^78^.

### Statistics and reproducibility

Each experiment was performed in triplicate at separate times, with similar results; representative data are shown.

## Supplementary Material

Supplementary Files

This is a list of supplementary files associated with this preprint. Click to download.

- video1.mp4
- 12TFvalidationreportnew.pdf
- combinedvalidationreportsupdated.pdf
- 7281471supp13132460thvd99NOW09062026.pdf
- video2.mp4

## Figures and Tables

**Figure 1:**
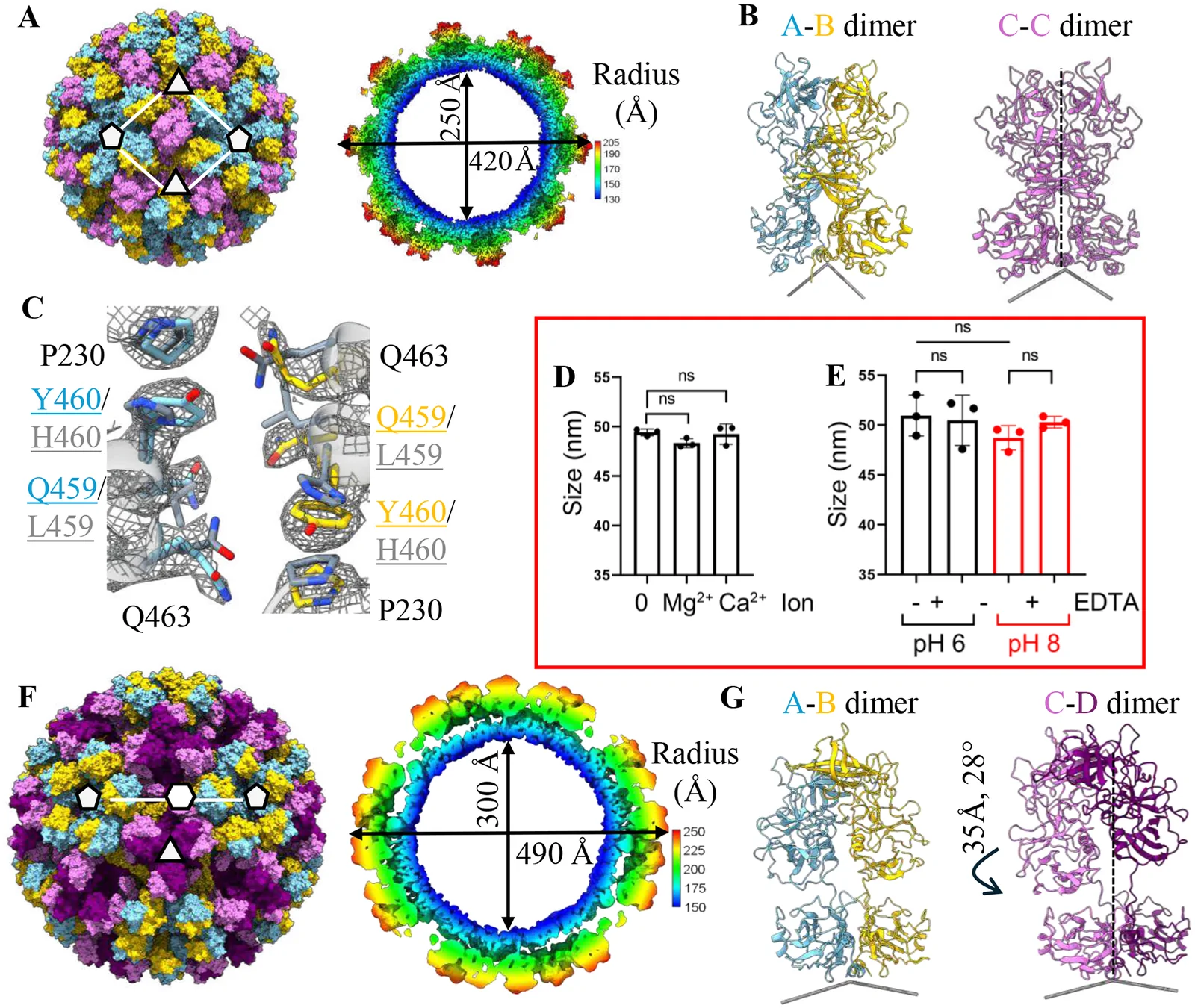
Cryo-EM structures of T=3 and T=4 VLPs from pandemic GII.4 Sydney. **A (Left)** Structure of T=3 VLP with quasi-equivalent subunits colored blue (A chains), gold (B chains) and violet (C chains). Icosahedral 5-fold axes are marked with pentagons and 3-folds axes with triangles. **(Right)** Cross-section of T=3 cryo-EM map colored by radius and showing VLP dimensions. **B** Quasi-equivalent subunits assemble into A-B and C-C dimers, viewed along 5→3-fold and 3→3-fold directions, respectively, with the 2-fold symmetry axis marked by a dashed line. The acute (A-B) or obtuse (C-C) angle of the shell is shown below the dimers. **C** Metal ion pocket in GII.4 HOV (grey, PDB: 7MRY) superimposed onto the corresponding GII.4 Sydney region. Amino acid differences are underlined. The cryo-EM map of GII.4 Sydney in this region is shown as a mesh and the residues are colored in grey (GII.4 HOV) or blue/gold (GII.4 Sydney). **D, E** DLS measurements of the hydrodynamic diameter of GII.4 Sydney VLPs with supplemental divalent cations **(D)** or with addition of EDTA in basic or acidic buffers **(E).** Significance was determined using two-tailed Student’s *t* test and is shown by ns = *p* **>** 0.05. **F (Left)** Structure of T=4 VLP with quasi-equivalent subunits colored blue (A chains), gold (B chains), violet (C chains) and purple (D chains). Icosahedral axes are marked as in **A**, and the quasi 6-fold axis is marked with a hexagon. **(Right)**. Cross-section of T=4 VLP colored by radius and showing VLP dimensions **G** In T=4, quasi-equivalent subunits form A-B and C-D dimers, viewed along the 5→quasi 6-fold and 6→quasi 6-fold directions, respectively. In both, the P-domains are lifted and twisted above the shell. Dashed line represents the true 2-fold axis from **B**.

**Figure 2:**
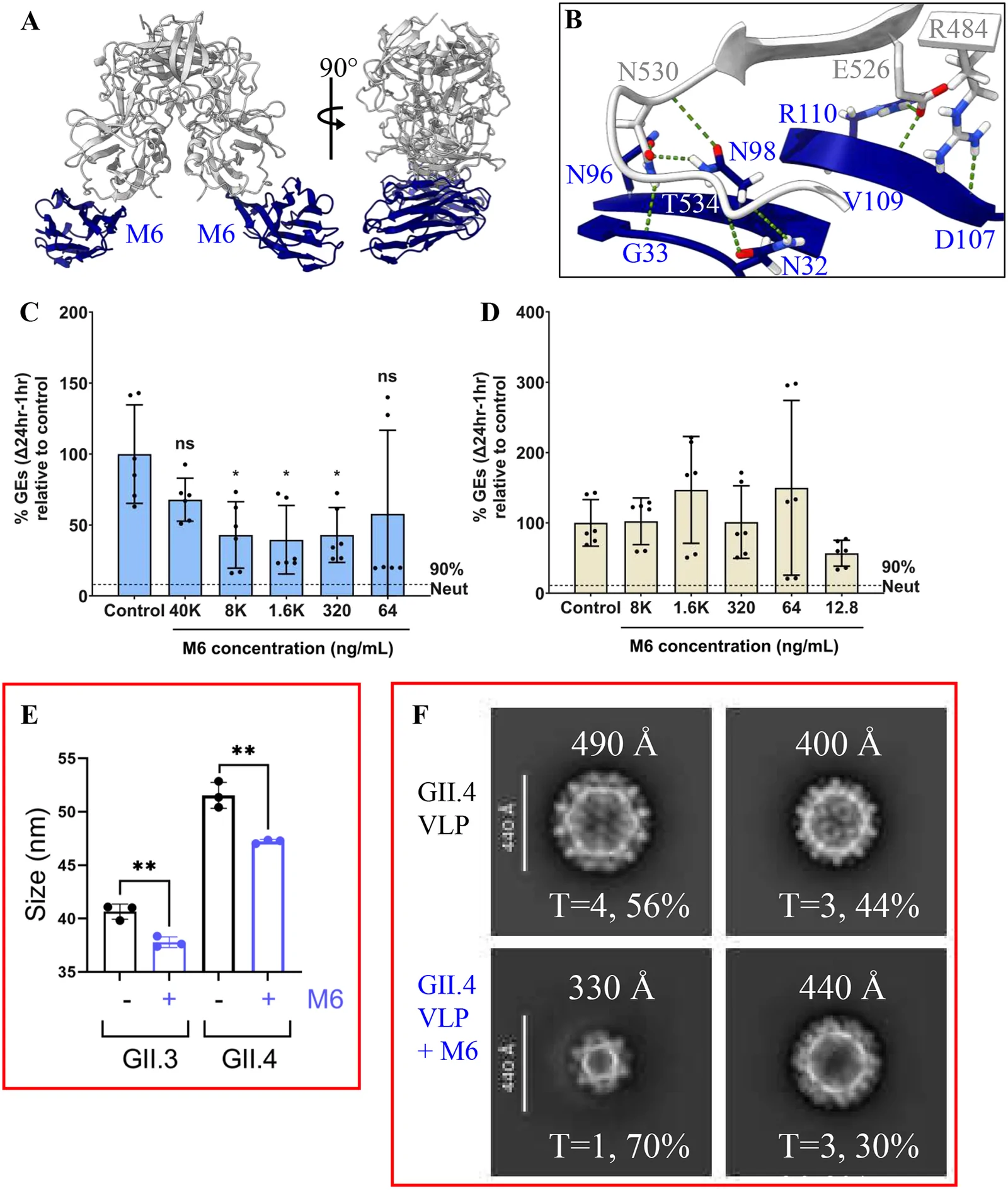
Binding of M6 to a cryptic epitope affects GII.4 Sydney capsid integrity and conformation. **A** The structure of a P-dimer (GII.3 genotype, colored grey) in complex with M6 (dark blue). **B** Close-up of the P-domain-M6 interface showing the sidechains of interacting residues as sticks. Green dashed lines= H-bonds. **C,D** Neutralization assays measuring GII.3 **(C)** and GII.4 Sydney **(D)** viral replication inside HIEs in the presence of different concentrations of M6. The assay quantifies the fold change in viral genome equivalents after 24 hr growth relative to an untreated control. Error bars denote the standard deviation and data bars represent the mean of n= 6 wells. Significance was determined using two-tailed Student’s *t* test and is shown by * = *p* < 0.05 and ns = *p* > 0.05. Dashed lines represent the expected values for achieving 90% neutralization. **E** Measurement of GII.3 and GII.4 Sydney VLP diameter with or without nanobody M6 by DLS. Error bars denote the standard deviation. Data points represent the peak from three rounds of measurements. Significance was determined using two-tailed Student’s *t* test and is shown by ** = p <0.01 **F** Representative 2D classes from cryo-EM datasets of GII.4 Sydney VLPs in the absence or presence of M6. Outer diameters and T numbers are displayed for each class, along with relative abundance.

**Figure 3:**
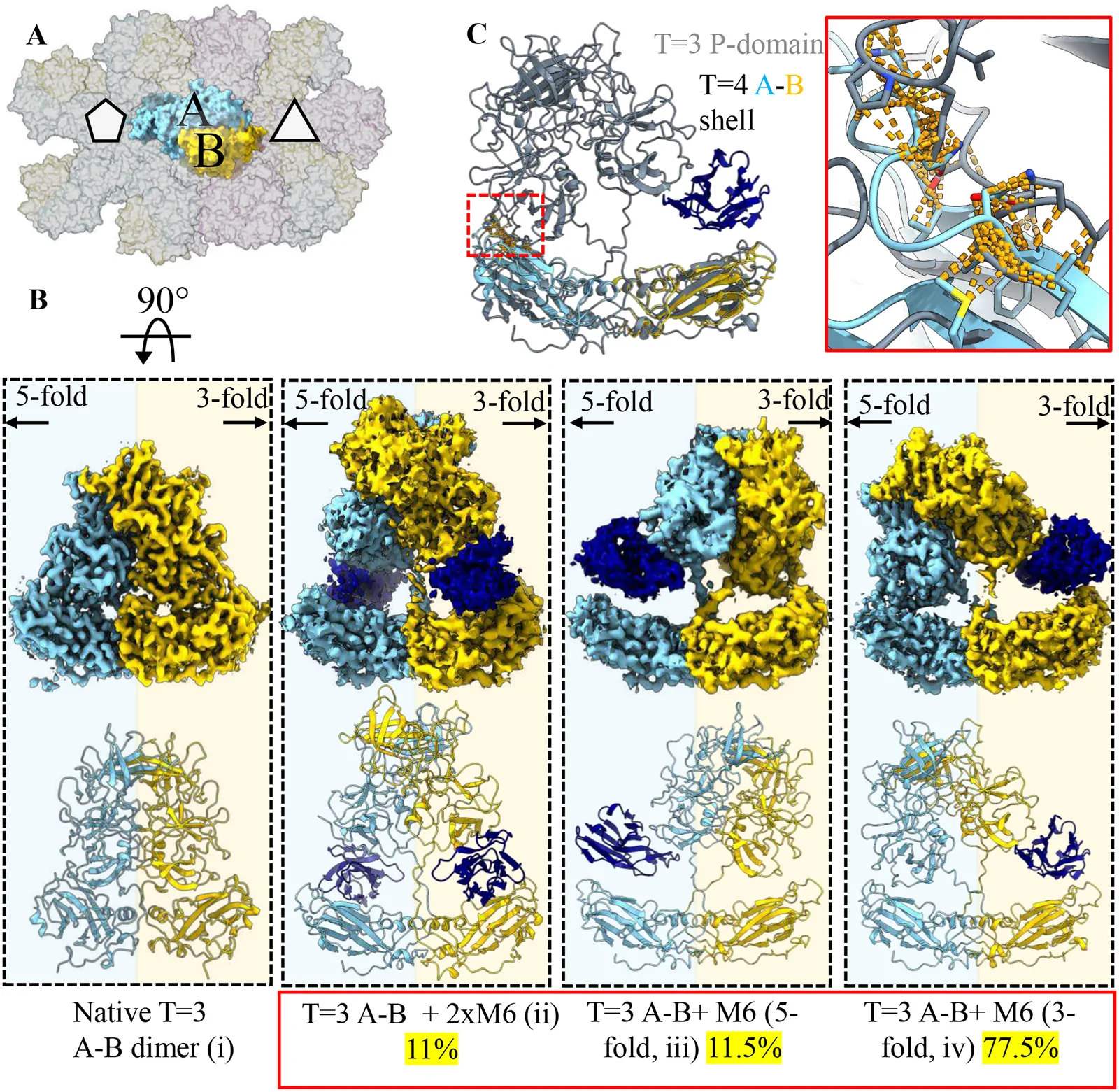
Conformational modalities of A-B dimers in response to M6 binding. **A** Top-down view of a T=3 A-B dimer (solid surfaces) and its placement within the local 5- and 3-fold symmetry axes (transparent surfaces). **B** Focus-refined 3D classes of M6-bound A-B dimers and their relative abundance. (i) Cryo-EM density (**top**) and cartoon representation (**bottom**) of A-B dimer in the native T=3 conformation shown for reference. (ii) A-B dimer with two M6 molecules bound simultaneously, (iii) with a single M6 bound from the 5-fold or (iv) from the 3-fold hollow. **C** Modeling of M6 binding in T=4 causes steric clashes based on the superposition of the S domains of M6-bound T=3 A-B dimer (grey) with the S domains of T=4 A-B dimers (blue and gold). **Inset:** Possible steric clashes (dashed lines) between the tilted P-domain and its corresponding shell arising from the larger angle between S-domains in T=4 VLPs. Color scheme: subunit A= blue, B= gold, M6= dark blue.

**Figure 4:**
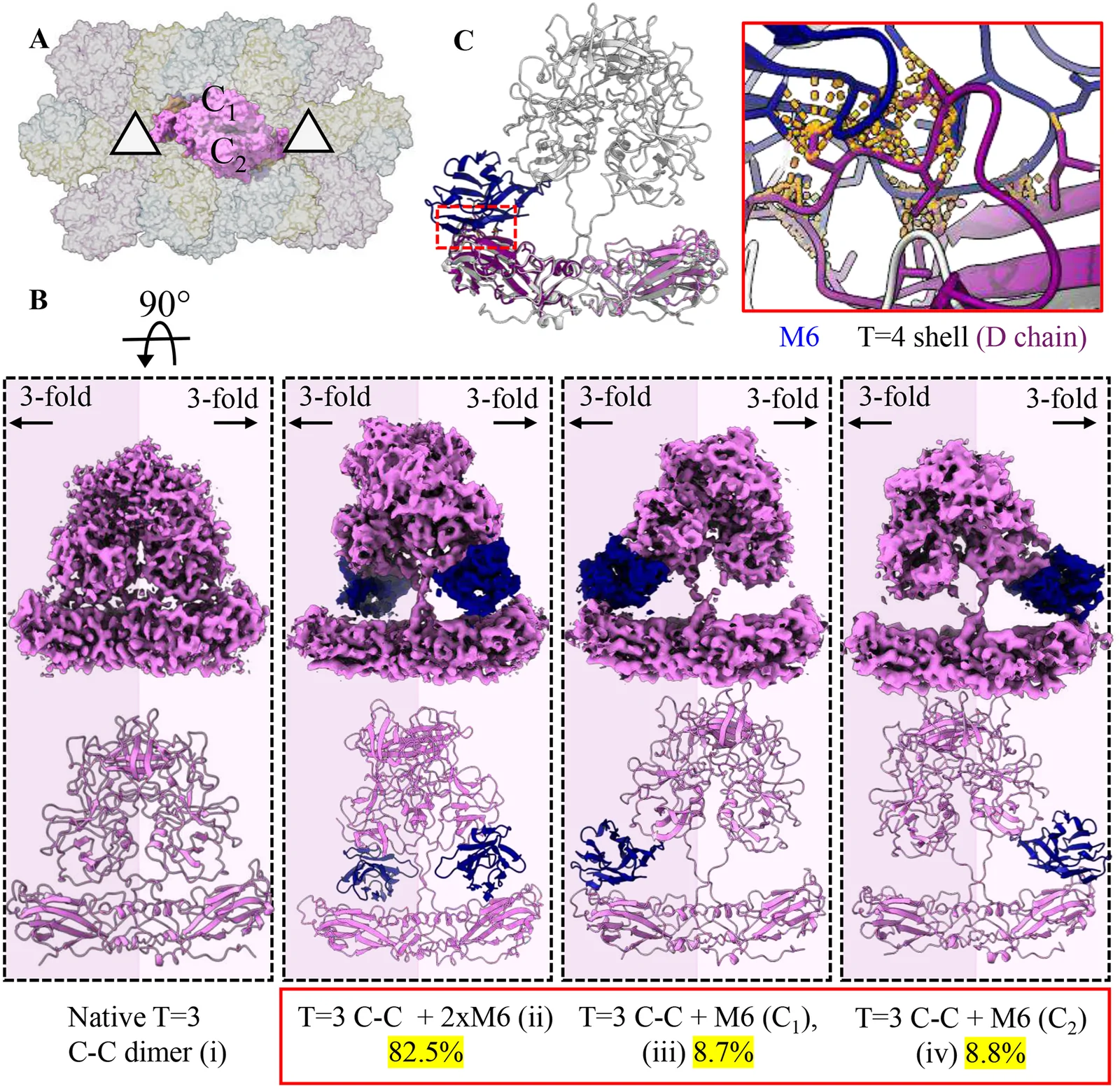
Conformational modalities of C-C dimers to accommodate M6 binding. **A** Top-down view of a T=3 C-C dimer (solid surfaces, here termed C_1_-C_2_ for clarity) and its placement within the local 3-fold symmetry axes (transparent surfaces). **B** Focus-refined 3D classes of M6-bound C-C dimers and their relative abundance. (i) Cryo-EM density (**top**) and cartoon representation (**bottom**) of C-C dimer in the native T=3 conformation shown for reference. (ii) C-C dimer with two M6 molecules bound simultaneously, (iii) with a single M6 bound to chain C_1_ or (iv) M6 bound to chain C_2_. **C** Modeling of M6 binding in T=4 causes steric clashes based on the superposition of the S domains of M6-bound T=3 C-C dimer (grey) with the S domains of T=4 C-D dimers (purple and violet). **Inset:** Possible steric clashes (dashed lines) between the nanobody and the shell domain nearest to its epitope interface arising from the larger angle between S-domains in T=4 VLPs. Color scheme: subunit C= violet, M6= dark blue.

**Figure 5:**
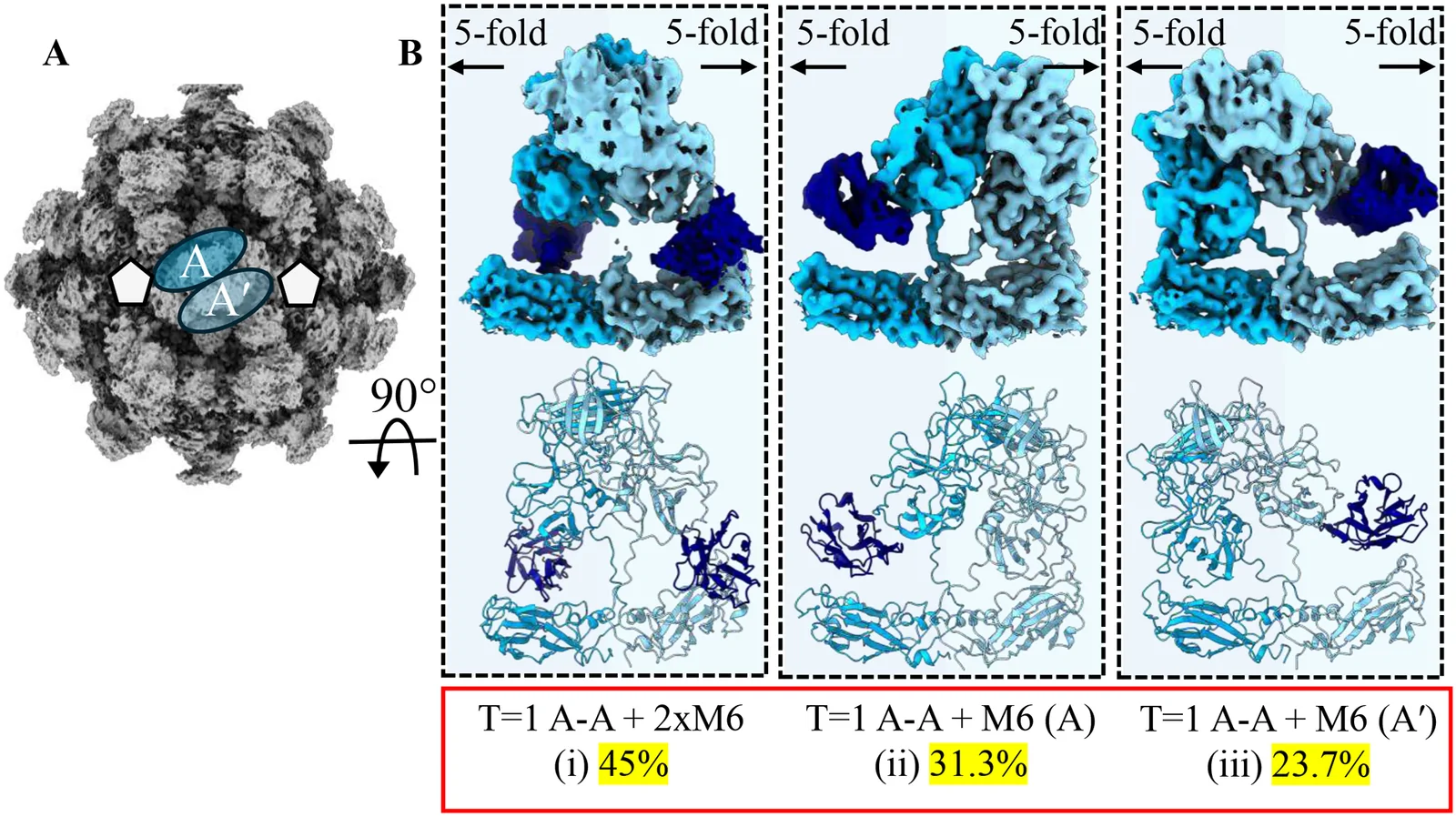
VP1 dimers from T=4 particles broken by M6 binding reassemble into pseudo T=1 VLPs. **A** M6-bound T=1 VLP map refined with strict icosahedral averaging, viewed along the icosahedral 2-fold axis formed by an A-A′ dimer. Icosahedral 5-fold hollows are marked with pentagons. **B** Focus-refined 3D classes of M6-bound A-A′ dimers and their relative abundance. (i) Cryo-EM density **(top)** and cartoon representation **(bottom)** of the A-A′ dimer with two nanobodies bound, (ii) with a single M6 bound to chain A, and (iii) M6 bound to chain A′. Color scheme: subunit A/A′ = light blue, M6= dark blue.

**Figure 6:**
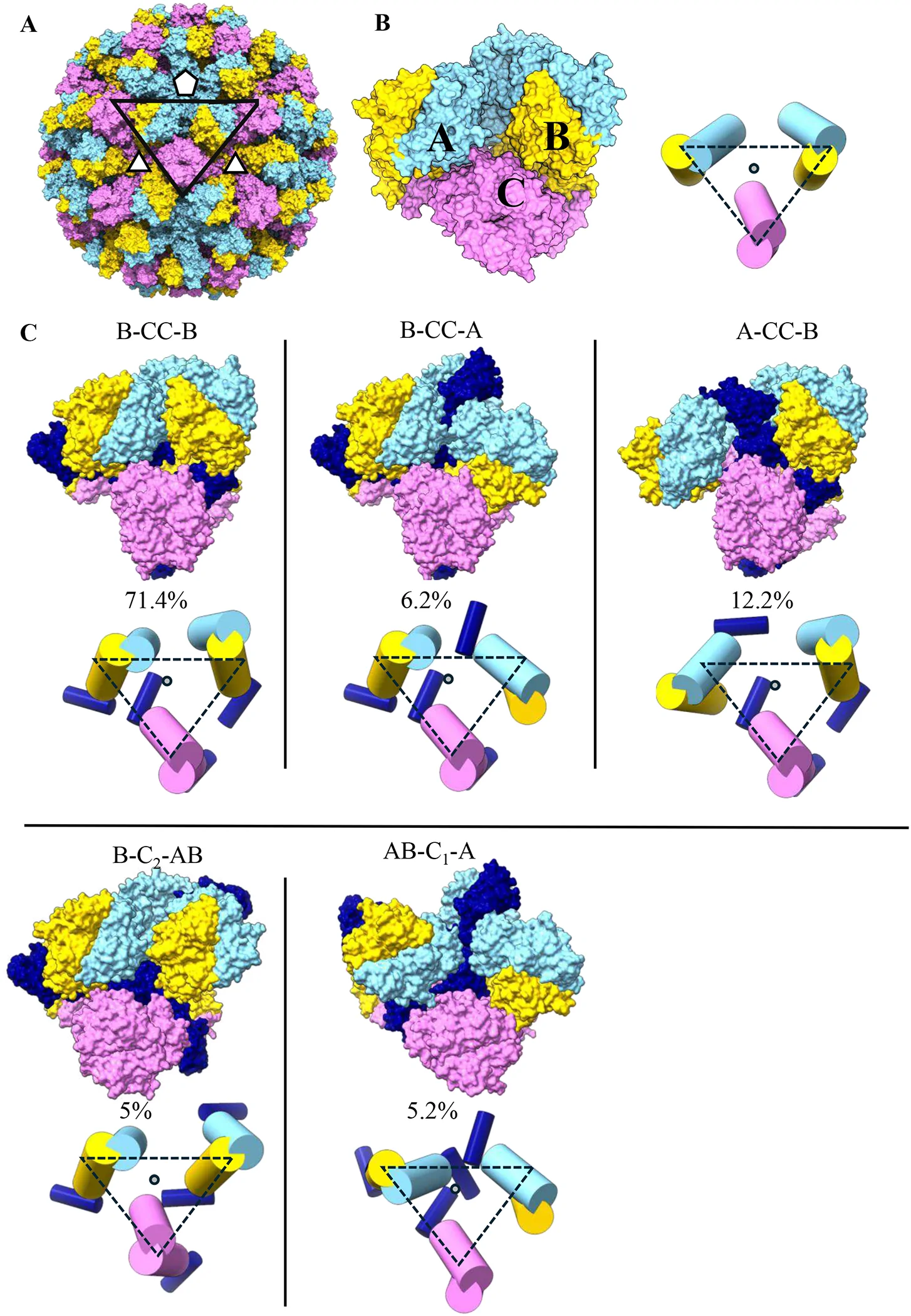
Cross-talk between adjacent M6-bound VP1 dimers in the capsid. **A** Overview of the untreated GII.4 Sydney T=3 VLP from Fig. 1A, with the 5- and 3-fold symmetry axes marked by a pentagon or triangles, respectively. The dimers inside the open triangle are shown in **B** in a close-up view. **B** The three labeled VP1 chains (A, B and C) forming an icosahedral asymmetric unit, and the chains that pair with them to form dimers, were extracted from the symmetry-expanded particle stack. Top-down view of the VP1 dimers surface **(left)** and simplified view showing each P-domain and their relative positions as cylinders **(right).** All cylinders were generated in ChimeraX1.11.1 between the Cα atoms of residues 333–520 for P-domains and residues 7–104 for nanobodies. Central dot = marker for placement of the dashed triangle, marking the corners of the 3 VP1 dimers in **B**. **C** Distinct M6 binding modalities revealed by 3D Classification with relative abundance of each class shown as a percentage. The structures from Figures 4–5 were fitted into the cryo-EM maps of each class. Cryo-EM densities are omitted for clarity. Classes are named after the VP1 chains that are bound by M6 in each dimer.

**Figure 7:**
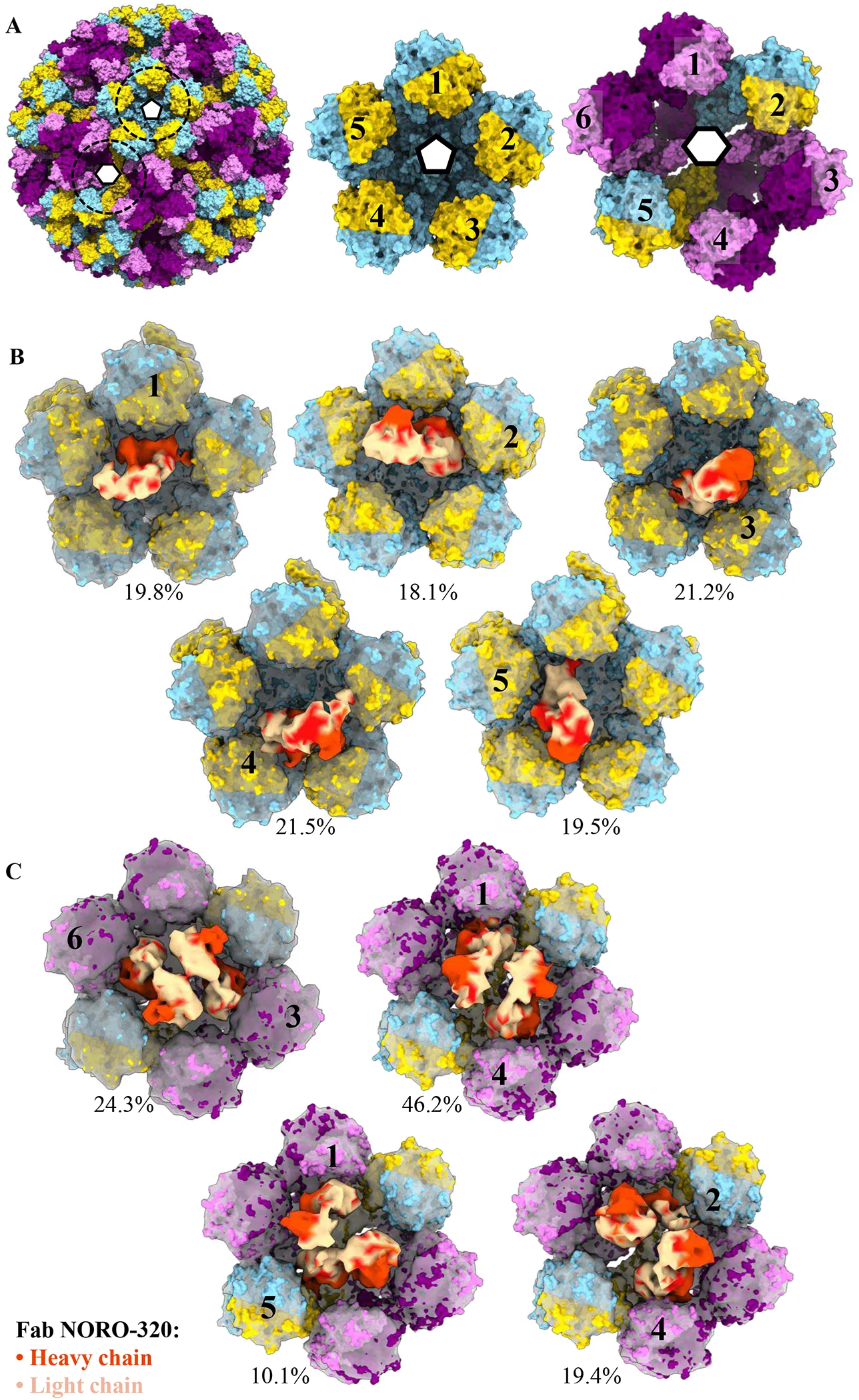
Human Fab NORO-320 covers all icosahedral hollows in mutually-exclusive conformations. **A** Overview of the GII.4 Sydney T=4 VLP from Figure 1F, with the 5- and quasi 6-fold symmetry axes marked by a pentagon or hexagon, respectively **(left).** The corresponding hollows are shown inside dashed circles and in close-up views on the **right**, with the VP1 dimers forming the hollows numbered for clarity. **B, C** 3D classes showing Fab NORO-320 binding modalities at the 5-fold **(B)** or quasi 6-fold **(C)** hollows, with relative distribution shown as a percentage. The Fabinteracting VP1 dimers are labelled, following the numbering scheme in **A**. The A-B and C-D dimers from a T=4 particle shown in Figure 1G were fitted inside the cryo-EM maps (transparent) with ChimeraX1.11.1, while the extra density corresponding to NORO-320 is colored orange.

**Table 1:** Cryo-EM data collection, reconstruction and refinement statistics for GII.4 Sydney T=3 and T=4 VLPs. The statistics of two confirmations of T=3 C-c dimers are also represented

Structure	GII.4 Sydney VLP		T=3, C-C dimer- local refinement	
	T=3 resting	T=4 raised	Class_1–3	Class_5–9
Accession codes	PDB: 12QZ, EMD-76702	PDB: 12TF, EMD-76740	PDB:13KF, EMD-77106	PDB: 13KG, EMD-77107
**Number of movies**	3290	3290	3290	3290
**Number of particles (total)**	30985	30985	13428	13428
**Number of particles (final**	13428	16898	205850	209178
**Defocus range (μm)**	0.5 to −2.5	0.5 to −2.5	0.5 to −2.5	0.5 to −2.5
**Electron dose (electrons/Å2)**	50	50	50	50
**Real-space model refinement**				
**Resolution range (Å)**	2.84 / 4.0	3.89 / 6.9	3.0 / 4.0	2.97 / 4.0
**Rwork / Rfree**				
**Number of residues**	540 (residues 1–11B and 1–24A/C missing)	540 (residues 1–11B and 1–24A/C missing)	540 (residues 1–21C and 1–22B missing)	540 (residues 1–21C and 1–22B missing)
**R.M.S. deviations**				
**Bond lengths (Å)**	0.003 (0)	0.006 (0)	0.003 (0)	0.004(0)
**Bond angles (°)**	0.503 (0)	0.739 (0)	0.526 (0)	0.555 (0)
**Ramachandran plot values**				
**Favored (%)**	94.14	91.7	97.53	96.73
**Allowed (%)**	5.79	8.3	2.47	3.27
**Disallowed (%)**	0.07	0	0	0
**Clash score**	4	23.8	2.77	2.46
**CC(mask)**	0.88	0.83	0.88	0.9

**Table 2:** X-ray crystallography data collection and refinement statistics.

Structure	GII.3 P-domain-M6
Accession code	PDB: 12PG
**Wavelength (Å)**	1.0002
**Resolution range (Å)**	45.24 – 2.2 (2.24–2.2)
**Space group**	P 61 2 2
**Unit cell**	
**a, b, c (Å)**	87.3, 87.3, 225.83
**α, β, g (°)**	90, 90, 120
**Unique reflections**	26770 (1307)
**Multiplicity**	30.0 (31.9)
**Completeness (%)**	100
**Mean I/sigma(I)**	14.8 (2.1)
**Wilson B-factor**	33.23
**R-merge (%)**	13.9 (78.5)
**R-work (%)**	20.55 (25.17)
**R-free (%)**	23.14 (27.55)
**Number of non-hydrogen atoms**	3483
**Macromolecules**	3300
**Ligands**	1
**Solvent**	182
**Protein residues**	425
**R.M.S. deviations**	
**Bond lengths (Å)**	0.003
**Bond angles (°)**	0.59
**Ramachandran**	
**Favored (%)**	97.37
**Allowed (%)**	2.63
**Disallowed (%)**	0
**Average B-factor**	41.45
**Macromolecules**	41.52
**Ligands**	32.55
**Solvent**	40.27

**Table 3:** Cryo-EM data collection, reconstruction and refinement statistics for GII.4 Sydney T=3 and T=1 particles bound to M6.

Structure	GII.4 Sydney+M6								
	T=3 raised, A-B dimer			T=3 raised, C-C dimer			T=1 raised, A-A’ dimer		
	5-Fold axis	3-Fold axis	2×M6	1×M6 (C1)	1×M6 (C2)	2×M6	1×M6 (A)	1×M6 (A’)	2×M6
Accession codes	PDB:12TW, EMD-76756	PDB: 12TX, EMD-76757	PDB: 12TY, EMD-76758	PDB:12TZ, EMD-76759	PDB: 12UA, EMD-76760	PDB: 12UB, EMD-76761	PDB: 12UC, EMD-76762	PDB: 12UD, EMD-76763	PBD: 12UE, EMD-76764
**Number of movies**	3283	3283	3283	3283	3283	3283	1967	1967	1967
**Number of particles (total)**	56958	56958	56958	56958	56958	56958	1834	1834	1834
**Number of particles (final reconstruction)**	104100	521498	99497	78998	79529	399802	525880	399653	755787
**Defocus range (μm)**	0.5 to −3.0	0.5 to −3.0	0.5 to −3.0	0.5 to −3.0	0.5 to −3.0	0.5 to −3.0	0.5 to −3.0	0.5 to −3.0	0.5 to −3.0
**Electron dose (electrons/Å2)**	50	50	50	50	50	50	50	50	50
**Real-space model refinement**									
**Resolution range (Å)**	3.39/5.5	2.93/4.6	3.44/7.3	3.44/7.0	3.21/7.0	3.03/6.5	3.16/5.0	3.2/5.0	3.19/5.0
**Rwork / Rfree**									
**Number of residues**	540 (residues 1–24 and1–12B missing)	540 (residues 1–25A and1–12B missing)	540 (residues 1–26A and1–12B missing)	540 (residues 1–24C, 1–25C’missing)	540 (residues 1–24C, 1–25C’missing)	540 (residues 1–9C and1–34C’ missing)	540 (residues 1–44A and 1–45, 114 A’ missing)	540 (residues 1–45A and 1–46, 114 A’ missing)	540 (residues 1–45A and 1–46, 114 A’ missing)
**R.M.S. deviations**									
**Bond lengths (Å)**	0.002 (0)	0.004 (0)	0.003 (0)	0.003 (0)	0.003 (0)	0.003 (0)	0.003 (0)	0.003 (0)	0.003 (0)
**Bond angles (°)**	0.6512(5)	0.616 (2)	0.663 (2)	0.544(4)	0.609 (2)	0.625 (0)	0.522 (3)	0.506 (0)	0.522 (6)
**Ramachandran plot values**									
**Favored (%)**	96.5	94.02	93.89	93.99	93.72	93.59	95.05	94.86	95.62
**Allowed (%)**	3.5	5.98	6.11	5.92	6.11	6.41	4.95	5.14	4.38
**Disallowed (%)**	0	0	0	0.09	0.18	0	0	0	0
**Clash score**	25.39	5.63	9.3	31	8.82	9.01	19.5	4.95	24.91
**CC(mask)**	0.81	0.83	0.67	0.76	0.81	0.7	0.83	0.83	0.76

**Table 4:** Cryo-EM data collection, reconstruction and refinement statistics for GII.4 Sydney T=4 and T=1 particles bound to Fab NORO-320.

Structure	GII.4 P12 Sydney + Noro 320						
	T=4 raised, A-B dimer		T=4 raised, C-D dimer			T=1 raised, A-A’ dimer	
	5-fold hollow	Quasi 6-fold hollow	1×Fab (C)	1×Fab (D)	2×Fab	2×Fab tilted left	2×Fab tilted right
Accession codes	PDB: 12TV, EMD-76755	PDB: 12TU, EMD-76754	PDB: 12TP, EMD-76749	PDB: 12TQ, EMD-76750	PDB: 12TR, EMD-76751	PDB: 12TS, EMD-76752	PDB: 12TT, EMD-76753
**Number of movies**	4683	4683	4683	4683	4683	4683	4683
**Number of particles (total)**	1403	1403	1403	1403	1403	6509	6509
**Number of particles (final reconstruction)**	12211	14568	10144	16421	22827	71634	72670
**Defocus range (μm)**	0.5 to −3.0	0.5 to −3.0	0.5 to −3.0	0.5 to −3.0	0.5 to −3.0	0.5 to −3.0	0.5 to −3.0
**Electron dose (electrons/Å2)**	50	50	50	50	50	50	50
**Real-space model refinement**							
**Resolution (Å)**	7.99	7.92	7.32	7.19	6.28	7.48	7.51
**Rwork / Rfree**							
**Number of residues**	540 (residues 1–40 A/B missing)	540 (residues 1–40 A/B missing)	540 (residues 1–44C/D missing)	540 (residues 1–44C/D missing)	540 (residues 1–44C/D missing)	540 (residues 1–40A/A’ missing)	540 (residues 1–40 A/A’ missing)
**R.M.S. deviations**							
**Bond lengths (Å)**	0.003 (0)	0.003 (0)	0.003 (0)	0.003 (0)	0.003 (0)	0.004 (0)	0.003 (0)
**Bond angles (°)**	0. 637(5)	0.588 (1)	0.671 (3)	0.705(16)	0.734 (3)	0.663 (19)	0.632 (2)
**Ramachandran plot values**							
**Favored (%)**	95.58	96.08	94.94	94.33	96.24	94.33	95.37
**Allowed (%)**	4.42	3.92	4.99	5.6	3.76	5.67	4.63
**Disallowed (%)**	0	0	0.07	0.07	0	0	0
**Clash score**	16.44	11.41	14.58	18.1	17.08	24.75	15.46
**CC(mask)**	0.59	0.56	0.74	0.76	0.71	0.64	0.75

**Table 5:** P-domain motions in the M6 and NORO-320 Fab-bound structures in relation to native VP1 conformations.

Structure	Rotation (°)	Lift (Å)	VLP type	Figure
A-B dimer + 2×M6	38	16	T=3	3
A-B dimer + M6 (5-fold axis)	34	11	T=3	3
A-B dimer + M6 (3-fold axis)	24	-	T=3	3
C-C dimer + 2×M6	40	18	T=3	4
C_1_-C dimer + M6	16	5	T=3	4
C-C_2_ dimer + M6	13	9	T=3	4
A-A dimer + 2×M6	32	14	T=1	5
A-A_2_ + M6	25	-	T=1	5
A_1_-A + M6	25	-	T=1	5
A-B dimer + NORO-320 (5-fold)	10	-	T=4	S7
A-B dimer + NORO-320 (quasi 6-fold)	10	-	T=4	S7

**Table 6:** Percentage of particles in each 3D class. Distinct 3D classifications are separated by thick borders.

Structure	Particles per class (%)	Figure
A-B dimer + 2×M6	11	3
A-B dimer + M6 (5-fold)	11.5	3
A-B dimer + M6 (6-fold)	77.5	3
C-C dimer + 2×M6	82.5	4
C_1_-C dimer + M6	8.7	4
C-C_2_ dimer + M6	8.8	4
A-A dimer + 2× M6	45	5
A-A dimer + M6 (A)	31.3	5
A-A dimer + M6 (A′)	23.7	5
B-CC-B	71.4	6
B-CC-A	6.2	6
A-CC-B	12.2	6
B-C_2_-AB	5	6
AB-C_1_-A	5.2	6
A-B dimer + NORO-320 (5-fold)	14.5	S7
A-B dimer + NORO-320 (6-fold)	17.4	S7
A-B unbound dimer	68.1	-
C-D dimer + 2× NORO-320	27.2	S7
C-D dimer + NORO-320 (left)	12	S7
C-D dimer + NORO-320 (right)	19.5	S7
C-D unbound dimer	41.3	-
A-A′ dimer + NORO-320 (left)	18.4	S7
A-A′ dimer + NORO-320 (right)	18.6	S7
A-A′ unbound dimer	63	-

## Data Availability

The atomic models built in this study have been deposited in the Protein Data Bank (PDB) and their related cryo-EM reconstructed density maps in the Electron Microscopy Data Bank (EMDB) as follows: PDB: 12QZ, EMD-76702 (T=3 resting GII.4 Syd VLP); PDB: 13KF, EMD-77106 and PDB: 13KG, EMD-77107 (T=3 resting GII.4 Syd C-C dimer confirmations), PDB: 12TF, EMD-76740 (T=4 raised GII.4 Syd VLP); PDB: 12TW, EMD-76756 (M6-bound A-B dimer 5-fold); PDB: 12TX, EMD-76757 (M6-bound A-B dimer 3-fold); PDB: 12TY, EMD-76758 (M6 double-bound A-B dimer); PDB: 12TZ, EMD-76759 (M6-bound CC dimer- C_1_); PDB: 12UA, EMD-76760 (M6-bound C-C dimer- C_2_); PDB: 12UB, EMD-76761 (M6 double-bound C-C dimer); PDB: 12UC, EMD-76762 (M6-bound A-A’ dimer- A); PDB: 12UD, EMD-76763 (M6-bound A-A’ dimer- A’); PBD: 12UE, EMD-76764 (M6 double-bound A-A’ dimer); PDB: 12TV, EMD-76755 (NORO320-bound A-B dimer 5-fold); PDB: 12TU, EMD-76754 (NORO320-bound A-B dimer quasi 6-fold); PDB: 12TP, EMD-76749 (NORO320-bound C-D dimer- C side); PDB: 12TQ, EMD-76750 (NORO320-bound C-D dimer- D side); PDB: 12TR, EMD-76751 (NORO-320 double-bound C-D dimer); PDB: 12TS, EMD-76752 (NORO320 double-bound A-A’ dimer- left tilted); PDB: 12TT, EMD-76753 (NORO-320 double-bound A-A’ dimer- right titled). X-ray crystal structure of GII.3 P domain bound to M6 has been deposited as PDB: 12PG.
